# Stakeholders perceptions regarding implementing maternal and newborn health care programs in Rwanda

**DOI:** 10.1186/s12913-021-06824-3

**Published:** 2021-08-11

**Authors:** Clemence Nishimwe, Gugu G. Mchunu

**Affiliations:** 1 School of Nursing and Public Health, University of KwaZulu-Natal, College of Health Sciences, King George Ave, Durban, 4001 South Africa; 2 Health Economics and HIV/AIDS Research Division (HEARD), University of KwaZulu-Natal, Durban, South Africa

**Keywords:** Maternal health, Newborn health, Maternal and newborn health program, Rwanda, Stakeholders

## Abstract

**Background:**

While maternal and newborn deaths has been decreasing since 2008 in Rwanda, there is room for improvement to meet its sustainable development goals. The maternal and newborn health care program needs to be monitored to ensure its effective implementation. This study therefore aimed to explore stakeholder’s perceptions of the Rwandan maternal and newborn health care program to identify areas for improvement.

**Methods:**

The convergent, parallel, mixed method study used quantitative and qualitative data in a single phase. The quantitative data was obtained from 79 health care workers, ranging from maternal community health care workers to program supervisors. The 10 areas of the Project Implementation Profile (PIP) instrument checklist with a five-point Likert scale were used to indicate their perceptions (strongly disagree to strongly agree). The qualitative interviews of five nurse managers used a manifest inductive content analysis, directed approach that entailed using existing theory and prior research to develop the initial coding scheme before starting data analyse.

**Results:**

There was disagreement about the level of top management support, human resources was regarded as an area of concern, with 18.7% (*n* = 14/79) indicating that they did not agree that this was adequately provided for; urgent solutions for unexpected problems was regarded as an areas of concern by 46.8% (*n* = 36/79). Top management support weakness were inadequate support training, materials, money for home visits, supervision and leaderships, and training of newly recruited maternity health care workers. For human resources, there were insufficient trained staff to take care of mothers and newborns due to the shortages of health providers. The management of unexpected problems was also an area of concerns and related to getting patients to health facilities during pregnancy emergencies and the lack of qualified birth attendants at health facilities.

**Conclusion:**

The study identified three areas for improvement: top management support, human resources and urgent solutions for unexpected problems, as they may be affecting the provision of maternal and newborn health care program services. Using the PIP enable managers to improve the country’s maternal and newborn health care program, and to provide ongoing monitoring and evaluation of with respect to the desired outcomes of reducing maternal and neonatal mortality.

**Supplementary Information:**

The online version contains supplementary material available at 10.1186/s12913-021-06824-3.

## Background

Globally, the number of annual neonatal deaths decreased from 5.0 million in 1990 to 2.5 million in 2018 [1], with efforts being made to implement programs that would enable countries to meet their Sustainable Development Goal (SDGs) targets (adopted in 2016) to reduce mortality to at least 12 deaths per 1000 births by 2030. In 2017, the majority of all neonatal deaths (75%) occurred during the first week of life, with approximately 1 million newborns dying within the first 24 h. Preterm birth, intrapartum-related complications (birth asphyxia or lack of breathing at birth), infections and birth defects caused most neonatal deaths [1], the majority of which (99%) occur in low- and middle-income countries, about half of which happen at home [1–3]. Sub-Saharan Africa had the highest neonatal mortality rate in 2018 at 28 deaths per 1000 live births, with a child born in the region being 10 times more likely to die in the first month than one born in a high–income country [1].

Of the women who die from causes relating to pregnancy, childbirth or postpartum [4], 99% (302000) occur in developing countries every year [5]. Sub-Saharan Africa (SSA) alone accounts for approximately 66% (201000) of maternal deaths, followed by Southern Asia (66000) [4]. Reducing maternal deaths globally to less than 70 per 100,000 live births by 2030 is a target of SDG 3, and aims to “ensure healthy lives and promote wellbeing for all at all ages” [6].

In Ethiopia, the maternal mortality ratio (MMR) ranges from 266 to 1667 per 100,000 live births (LB) [7], the causes being multi-factorial, with demographic, behavioral, nutritional and health services factors being related to poor maternal health outcomes [7]. The causal factors for the death of mothers include those associated with poor health-provider competence, low number of health facility deliveries, inefficient referral systems for obstetric emergencies and lack of emergency obstetric services at facilities [7]. The neonatal mortality rate was 37 per 1000 live births in Ethiopia in 2011, compared to 35.9 per 1000 live births in Africa, a slow decline having been achieved with considerable effort from the government and other stakeholders [8]. A study reported that the main predictors identified for an increased risk of neonatal mortality were neonates from rural areas, birth order of greater than five, home delivery, very low and low birth weight, and the inability to cry at birth [8].

In Kenya, the maternal mortality ratio was 362 deaths per 100,000 live births and neonatal mortality rate 22/1000 live births in 2017 [9], with free delivery services being found to be an important strategy to promote the utilization of health facility delivery services, with the need to simultaneously address other factors that contribute to pregnancy mortality in the country [9]. South Africa’s maternal mortality rate declined from 189 deaths per 100,000 live births in 2009 to 135 deaths in 2016 [10], with the neonatal mortality rate being 11.5 deaths per 1000 live births, in 2019 [11].

In Rwanda, the rates of newborn mortality have been slow to decline, and constitute approximately one-third of all child mortality [12, 13], with reproductive, maternal, newborn and child health remaining a high priority [14]. The Rwanda Demographic Health Survey (RDHS) 2014/2015 reported that the neonatal mortality rate was 20 deaths per 1000 live births and maternal mortality rate was 210 per 100,000 [15]. The rates were slow to decline, with the neonatal mortality rates (per 1000) being 44 in 2000, 37 in 2005 and 27 in 2010, and the maternal mortality ratio (per 100,000) being 1071 in 2000, 750 in 2005 and 476 in 2010. The Millennium Development Goals (MDGs) by 2015 for the neonatal mortality rate (per 1000) was 12 and maternal mortality ratio (per 100,000) 268 [13]. Rwanda is not close to achieving their SDGs targets of less than 70 maternal mortalities per 100,000 live births, but is on its way to achieving less than 12 neonatal deaths per 1000 live births by 2030 [13]. Increasing the coverage of maternal and neonatal health services is one of the goals of the Rwandan Ministry of Health, the intention being to improve the demand for and access to quality care to ensure universal coverage towards realizing the country’s SDGs [15].

Newborn deaths remain higher than desired (16.3 deaths per 1000 live births, in 2018) in Rwanda [16], with the Maternal, Neonatal and Child Health (MNCH) National Strategic Plan having set its targets as a framework to address this issue [15, 17]. MNCH interventions are being implemented in health facility and communities, such as family planning, maternal and newborn care, case management and nutrition. As part of a coordinated approach, maternal death surveillance and response, confidential survey for maternal deaths, stillbirth and birth asphyxia audit, and the scale-up of verbal autopsies countrywide were implemented to reduce maternal, newborn and child avoidable deaths. The use of cellphone Rapid SMS technologies supports the monitoring of the MNCH intervention implementation [15, 17], the various measures being intended to considerably reduce maternal, neonatal and child morbidity and mortality between 2018 and 2024.

Pinto and Slevin [18] identified 10 critical success areas for a Program Implementation Profile (PIP), this being a useful tool that can be adapted for program managers to understand the effectiveness of the programs they are implementing, including monitoring maternal and newborn health (MNH) care initiatives. The current study focused on one component of the community health packages, namely: mother and newborn health care program, and entailed exploring the health care providers’ perceptions of its implementation to identify possible weaknesses that could be improved. The research question was: what critical success factors affect the implementation of the maternal and newborn health care program, these relating to 10 areas that are regarded as being important for program success, as detailed below. Specific health workers were invited to participated in the mixed methods study, these being maternal health care workers (MCHWs) nurse and data manager, staff working in maternity wards (nurses and midwives), health facility and district hospital supervisors of MCHWs. This study therefore aimed to explore the health worker’s perceptions of the implementation of the Rwandan maternal and newborn health care program to identify areas for improvement.

### Description of maternal and newborn health care program

The maternal and newborn health program targets are in line with Rwandan Health Sector Strategic Plan 4 to address the maternal, neonatal and child challenges facing the country. The program started in 2008, when the National Roadmap was adopted to campaign for the accelerated reduction of maternal, newborn and child mortality in Africa (CARMMA), an initiative by the African Union (AU) Commission to address the associated challenges. The CARMMA is derived from the key priority areas enshrined in the AU policy framework for promoting sexual and reproductive health and rights in Africa (2005) and the Maputo plan of action (2006). In May 2009, the AU launched the CARMMA during the 4th session of the conference of AU Ministers of Health held in Addis Ababa, Ethiopia, to trigger concerted and increased action towards improving maternal and newborn health and survival across the continent. At the 5th conference of Ministers of Health in 2011 it was agreed that the CARMMA should be expended to address newborn and child health [19].

In Rwanda, the maternal and the newborn health program includes the roadmap (national roadmap to accelerate the reduction of maternal and infant mortality) that outlines approaches to reduce mortality, as well as strategies for improving the quality of facility-based primary and referral care, provide kangaroo mother care (for low birth weight infants in health facilities intervention) and community-based services for women during and after pregnancy. The home-based maternal and neonatal health care package (HB-MNCP) was adapted as a strategy to reduce maternal and neonatal deaths, and is a part of the program [20].

### Critical success factors for the maternal and newborn health care program in Rwanda

Pinto and Slevin [18] identified 10 critical factors that need to be explored for project success, and were applied in terms of their application to the HB-MNCP program in Rwanda: project mission, top management support, program schedule/plan, client consultation, human resource, technical tasks, client acceptance, monitoring and feedback, communication and urgent solutions for unexpected problem. Each will be discussed with respect to its relevance to this study.

#### Factor 1: project mission

The project mission and goals need to be clear, specific, operational, and understandable [18]. Rwanda’s HB-MNHCP program aims to improve women, newborn and child health by providing an environment where unwanted pregnancies are avoided, women can go through pregnancy and childbirth safely, their newborns are born alive and healthy, and children grow and develop to their full potential [15].

#### Factor 2: top management support

This is required to gain the necessary resources to adequately support a project through to completion [18]. In Rwanda, the government leads the implementation of the ‘roadmap’ with the support of partners and the international community, with a financial component included in the health sector financing plan [15]. The Ministry of Health (MoH) covers all costs for staffs, forms, registers and routine supervision, with a cost analysis of the program being necessary to quantify the resources required for implementation [20]. The top management need to ensure the payment for training, material development and printing costs, and most of the costs of the MCHW equipment and supplies [20].

#### Factor 3: program schedule/plan

All the activities needed to ensure successful program implementation should be scheduled, and the necessary people, money, time and other resources allocated. Furthermore, it must include a workable plan and assess the implementation process against the scheduled expectations [18]. In Rwanda’s case, the criteria for assessing the HB- MNCP are the core guidelines for the program, namely: identify pregnant women; conduct home visits during pregnancy; refer pregnant women for antenatal care (ANC); screen mother and newborns at the same time, and conduct home visits during the postnatal period. The training package includes core postnatal care (PNC) competencies for community health workers (CHWs) who provide PNC home visits, which requires delivering key maternal and newborn interventions [20]. Each MCHW receives a kit containing essential equipment and supplies, with referral notes being given to all women and babies referred to facilities. Community coordinators at the district hospital level, and community health worker supervisors at the health facility level, are essential for planning, training and supporting the MCHWs [20]. Women are advised to deliver at a health center, with those who do so at home being sent for PNC within 24 h of delivery, often being accompanied by their MCHW [20]. PNC may also be achieved through childhood immunization sessions and outreach visits [21]. The supervisory guidelines recommend visits by community supervisor at the district hospitals to the community supervisors at the health facilities every 4 months [20], 3 days training on supervision and an the use of an integrated supervisory checklist. The costs for the supervisors are covered by the district MOH funds, with a general budget for all supervisory activities, not only those that are community-based [20].

#### Factor 4: client consultation

This entails including persons who will be the project recipient or participant, and could be persons outside or within a related health department [18]. In this study, client consultation relates to engaging the people involved in utilizing the Primary Health Care (PHC) services that are linked to the community based maternal and newborn health program (CB-MNH). These include the mothers and family members who assist them, those who access antenatal care, delivery, emergency obstetric care (EMoC) services, manage sick newborn and PNC home visit situations.

#### Factor 5: human resource

It is important to include adequate personnel in a program, ensure their training, associated infrastructure and continuing professional development (CPD). There need to be criteria for selecting, recruiting, developing and retaining MCHWs and trained health providers [22]. Sliven and Pinto [15] emphasized that attention should be paid to selecting and training key personnel who can ensure project success. In Rwanda, key personnel are skilled birth attendants, especially midwifes, with at least one midwife in a maternity ward being required [12].

#### Factor 6: technical tasks

Successful project implementation needs to be managed by skilled people who understand the program and are able to implement or use the required technology [15]. The Rapid SMS-MNCH system makes it possible for effective and real-time two-way communication between MCHWs at the community level and those involved in the rest of the system, such as ambulance and health facility staff at the district and central hospitals. The primary expected results of the system are improved access to antenatal and postnatal care, institution delivery and emergency obstetric care. The MCHWs use cell phones, with staff (data managers) being needed to support them to receive and send messages, and compile their monthly reports.

#### Factor 7: client acceptance

The use of the health facility services needs to be negotiated with the clients [15], these being the mothers, maternal health workers and health providers in Rwanda. Client consultation does not assume their acceptance of any program, with ongoing monitoring being needed to establish their uptake and satisfaction with the services. In Rwanda, client acceptance of the health facility by the community regarding the maternal and newborn health care program is established by community utilization of the ANC, delivery, PNC, emergency and other health facility services. It includes the immunization levels, and the mothers working with and taking the advice of the MCHW and other health staff regarding their own and their newborns’ care [21].

#### Factor 8: monitoring and feedback

This refers to the project control processes, and entails keeping abreast of how a project is progressing compared to initial projections at each stage of its implementation and thereafter. Monitoring and feedback refers not only to observing project schedules, processes and budgets, but to the performance of members of the project team, and providing feedback about what works and what does not to enable changes to be made as needed [10]. This entailed the analysis of the monthly monitoring system, and is related to the use of the data for integrated planning and supervision at the district level, the intention being to improve the referral system at community and facility levels [23]. The MCHWs complete community registers for each pregnant woman, with the data being entered onto summary forms each month and submitted to the health facility community supervisor for review [20].

#### Factor 9: communication

Two-way communication is essential within and between the project teams, the organization and the client, and requires information about the project being available to all levels of operation. Communication must be clear within the project team, and in accordance with the goals and objectives of the project. The team members need to have formal channels of communication and regularly provide written status reports [15]. In Rwanda, the SMS alert system approach is also used to send data to the community health desk, the MCHW information system at various levels of the Ministry of Health about new pregnancies and complications, ANC visits made, facility appointments and delivery due dates [20]. The MCHWs are provided with mobile phones, a charging device and cellphone airtime to enable them to communicate regarding the following-up of mothers and babies at community level with the health facilities, and with ambulance drivers in case of emergency with short messages (SMS) [13].

#### Factor 10: urgent solution for unexpected problem that arise in the program

This is the ability to urgently address any unexpected problems that might arise that requires each team to have technically competent people with specific roles that are supported by effective systems and processes [15]. In Rwanda, it relates to identifying solutions when problems arise both before and after facility deliveries, with facility staff being required to send mothers back to their villages with referral forms for the MCHWs to ensure that they are aware of their return and continue to make home visits [20]. The MCHWs provide mothers and newborns identified with suspected danger signs are given referral slips to attend the health facilities for further investigation [20]. Community ambulances are available for referral but their reach remains relatively limited. Success in terms of being able to respond urgently requires there to be adequate transport, accessible roads, the availability of mobile phones and a working network for message to be sent and received [20].

These 10 critical success factors were investigated in this study to establish the health workers perception of the effectiveness of its implementation, and could be adapted for the maternal and newborn health care program and used as the routine tool of monitoring and evaluation.

## Methods

### Study design

This study entailed the use of quantitative and qualitative techniques within a convergent, parallel mixed method methodology that requires both quantitative and qualitative data to be collected during a single phase of research [24]. Mixed methods compensate for the weakness of each type, but with the different methods remaining autonomous, operating side-by-side. This approach prioritizes the two methods equally, keeping the data analysis independent but combining the results during the overall interpretation, using both to look for convergence, divergence, contradictions, or relationships from the two data sources using data triangulation [24]. The purpose of the convergent design is to obtain different but complementary data (quantitative and qualitative) on the same topic to best understand the research problem [24]. Holloway and Wheeler (2013) defined qualitative data collection as a form of social inquiry that focuses on the way people make sense of their experiences and the world in which they live [25]. In this study, the qualitative research approach focused on interviews [26], while the quantitative component entailed the participants completing the PIP questionnaire.

### Study setting

The Rwandan public health system consist of four levels: community, health facilities, district hospitals and national level hospitals being the final referral point, with the health system being decentralized to the district level. Rwanda has five provinces, with three public sector districts hospitals being purposively selected from 30 within the three of the provinces of Kigali City, Eastern Province and Western Provinces, and two each of their urban, semi-urban and rural associated health facilities being selected for inclusion. The health facilities were select based on their accessibility, these being: Kigali City Province: Muhima District Hospital, Muhima and Butamwa Health facility (urban); Eastern Province: Nyamata District Hospital, Nyamata and Mayange health facilities (semi-urban); Western Province**:** Kibogora District Hospital, Kibogora and Nyamasheke Health facility (rural).

### Study population, sampling and data collection instruments

The participants consisted of maternal community health workers (MCHWs) and all staff working in the maternal and newborn health care programs at health facilities and district hospitals, namely: nurses and data managers, the nurses and midwives in the maternity ward and immunization services, and the district-based community health worker supervisors. The nurse managers provide information about the implementation and integration of maternal and newborn health care program and are responsible for the MCHW training and overall supervision. The data managers are responsible for patient records kept and training the MCHWs to record and send data to the health facilities. The nurses and midwives provide PNC, delivery and ANC services to the women in their catchment communities. The district level supervisors MCHW supervisor are community coordinators who oversee the health facility-based MCHW supervisors.

Non-probability convenience sampling was used to identify 100 individuals to participate in the quantitative PIP questionnaire. The staff at each of the six sites consisted of those working in the program (MCHWs, nurses and midwives, nurse and data managers, community supervisors) with 30% of the MCHWs being included out of a total of 224 in the three selected districts. The team in charge of the maternal and newborn health care program was purposively selected from each health facility or district hospital according to their availability during working hours. These consisted of 30% of the MCHWs, one nurse manager, one data manager, and two nurses and/or midwives in the maternity wards, and one district hospital level community supervisor. All those who provided informed consent completed the PIP questionnaire.

Qualitative research typically involves purposeful sampling to enhance the understanding of the information–rich case [27]. It is oriented toward the development of ideographic knowledge from generalizations about individual cases. The six health facility nurse managers were invited to participate in the semi-structured interviews, as they could provide rich information regarding the implementation of the maternal and newborn health care program based on their educational background, management and supervisory experience in the various clinical settings. However, one nurse manager was unavailable, which resulted in five participants.

A meeting was held with the selected staff at each of the six health facilities to outline the project, establish their willingness to participate and complete a written informed consent form, which they were given a copy of for their records, with standard ethical considerations being observed throughout the study. Those who provided consent were asked to complete the questionnaires, which took approximately 30 min, and were provided with assistance where required.

Three data collection tools were used, the first being to obtain their demographic details on a standardized questionnaire, the second being the Project Implementation Profile (PIP) instrument to monitor the current perceived state of each of the 10 successes areas with all the participants (Appendix, Supplementary file 1, point 4). They were asked to indicate the extent to which they agreed with statements about factors affecting the implementation of the maternal and newborn health care program using a five-point Likert scale, ranging from ‘strongly disagree’ to ‘strongly agree’.

The third was a question schedule to obtain qualitative data from semi-structured interviews with the five nurse manager participants who had completed the questionnaires, the interviews being audio-recorded and transcribed for interpretation using a manifest content analysis directed approach (Appendix, Supplementary file 2). The directed content analysis used prior research and the PIP areas of interest to develop the coding scheme. The quantitative cross-sectional descriptive and qualitative methods entailed establishing the extent to which the stakeholders perceived the 10 statements to accurately reflect the status of the maternal and newborn health program:

1. ***Program mission***: Initial clarity of goals and general directions;
2. ***Top management support***: Their willingness to provide the necessary resources and authority /power for project success;
3. ***Project schedule /Plan***: A detailed specification of the individual action steps required for implementation;
4. ***Client consultation***: Communication, consultation and active listening to all impacted parties;
5. ***Human Resources***: Recruitment, selection and training of the necessary personnel for the project team;
6. ***Technical tasks***: Availability of the required technology and technical steps to accomplish specific actions;
7. ***Client acceptance***: The act of ‘selling’ the final project to its intended users and their uptake of services;
8. ***Monitoring and feedback***: Timely comprehensive control information at each stage of the process and feedback mechanisms to make modification as required;
9. ***Communication***: An appropriate network and necessary data to all key persons in the project implementation;
10. ***Urgent solution for unexpected problem arises in the program***: Ability to handle unexpected crises and deviations from the plan, and develop solutions that focus on implementing the components of the program [18].

### Validity and reliability

Validity is linked to the question of quality, with the value and wholeness of the work being key elements of validity judgements that ensure that the data can be trusted [28]. The instrument or procedures used in the research must be trusted to measure what they supposed to measure [29]. For the quantitative aspect of the study, a pilot study was conducted using 10 participants to test the data collection instruments for internal validation and reliability to ensure that the researcher obtains the appropriate data, with feedback being used to modify the questions. Some questions were adapted from the literature review and the Rwanda Health Sector Strategic Plan 4 to specifically address the maternal, neonatal and child mortality.

In addition, the study included the 10 areas of the Project Implementation Profile (PIP), with permission to use the instrument being given by the Project Management Institute (PMI). The PIP instrument was initially validated as a useful diagnostic tool for practicing manager and adapted according to the program under investigation in this study [30]. In the current study (Appendix, Supplementary file 1), the Cronbach’s Alpha was .76, which was an acceptable internal consistency as it was above the 0.70 indicated by Polite and Beck (2012), was considered to be acceptable [31].

### Trustworthiness of qualitative data

The trustworthiness of a qualitative study can be increased by maintaining high credibility and objectivity [25]. This was addressed by issues of credibility, which relates to the notion of internal validity, and entailed ensuring that the participants’ responses were accurately reflected. Through prolonged engagement in the setting, the researcher reflected on how she may have influence data collection, analysis, interpretation and write up. The researcher has completed a Masters in Nursing (Community Health specialisation), experience in the settings that was researched, and in the field of the maternal and newborn health care program performed by MCHWs in the community. The participants are experienced in the field of maternal and newborn health and gave their opinions about their experiences, with the researcher‘s findings being a reflection of the perceptions of the population under study [25].

### Data collection process and analysis

The participants provided demographic data from a questionnaire and indicated their opinions about the critical success factor of the Maternal and Newborn health care program through the PIP questionnaire. The quantitative data was analysed using various statistical measures that included a needs assessments and empowerment analysis. It provided an opportunity for a mixed methods study to contribute to learning about what worked regarding the implementation of the program as well as to explore its effectiveness in achieving the desired maternal and newborn health outcome [24].

The participants were asked to indicate their perceptions about the 10 key performance areas of the PIP regarded as important in maternal and newborn health care programs. The five-point Likert scale answers ranged from 1 = strongly disagree [SD], to 5 = strongly agree [SA]. The mean of each statement was out of 5, with the scales being grouped into three categories of (1) disagree, (2) Neutral (3) and agree. The data was analysed using the statistical package for Social Sciences (SPSS 25.0), with a mean value of four being required to be regarded as an indicator [32].. The questionnaires were numbered and coded to facilitate data capturing and auditing, and to ensure data confidentiality.

The qualitative analysis entailed content analysis, which required identifying codes, with the initial categories being revised and refined with ongoing analysis. A directed approach was used for the 10 PIP areas in the context of study, hence that used for qualitative data being directed content analysis [33]. This process of analysis goes through various stages: transcribing interviews and sorting field notes; organising, ordering and storing the data; listening to and reading or viewing the material collected repeatedly [25]. In this study, the computer-aided programme 12 Nvivo was used to organise and analyse the qualitative data.

The qualitative analysis entailed the following analysis processes:

(1) Transcribing interviews and sorting field notes: a combination of audio-taping of participants responses and field notes for observations were used during the individual interviews.

(2) Ordering and organising the data: the recorded material was cross checked and labelled for final analysis, with the material being stored in appropriate files for later retrieval.

(3) Analytical steps: this entailed a variety of analysis measures, such as listening to, viewing and gaining a holistic view of the data, as well as dividing them into units or segments of meaning. The analysis steps were: reflect on each transcript and search for significant statements; record all relevant statements; delete repetitive and overlapping statements; leave only invariant constituents of the phenomenon; organise, link and relate these into themes, including verbatim quotes from the data; integrate the themes into a description of the texture of the experience as told by the participants; reflect on this and their own experiences; and develop a description of the meanings of the experience [25].

(4) After obtaining written meaning of their experiences, the researcher returned to the supervisor for a review of the analysis process.

The point of interface for the mixed methods entailed the convergent parallel design collecting and analysing two independent strands of quantitative and qualitative data at the same time in a single phase. It prioritizes the methods equally, keeps the data analysis independent, combines the results during the overall interpretation in an effort to find convergence, divergence, contradictions, or relationships of the two sources of data [24].

Triangulation, which can combine quantitative and qualitative methods, was used as some of the participants who completed the questionnaire were interviewed, with the answers from the two data sets being compared [25]. Data triangulation was used to ensure that the inherent bias of one method was countered-balanced by the strengths of other. Using mixed methods is useful to establish if the results converge or corroborate one another, strengthening the validity of the findings. Triangulation seek convergence, corroboration and correspondence of results from different methods [25].

### Ethical considerations

Ethical considerations, which are related to the protection of the rights, dignity, safety and well-being of human subjects, underpinned this study. According to Brink (2006), a researcher is responsible for conducting research in an ethical manner and must have paid attention to all requirements [30]. The mayors of the Bugesera, Nyamasheke, Nyarugenge Districts provided gatekeeper permission, and confirmed that were in support that the health facilities being used should the university ethics committee approve the study. Approval for the study was obtained from the Biomedical Research Ethics Committee (BREC) at the University of KwaZulu-Natal (BREC Ref No: BE029/18), South Africa, and the Rwanda National Ethics committee (RNEC) (No.182/RNEC/2018).

According to Emmanuel, Wendler, Killen and Grady (2016), it is important to subscribe to ethical standards [34], with the following issues being addressed:

#### Protecting anonymity and confidentiality

The anonymity of the target population was ensured by not having any personal identification attached to the data collection responses. Participants only agreed to volunteer if sensitive information was held in confidence. For the questionnaire, confidentiality was guaranteed by using coding and storing the completed forms in a locked cupboard in a locked room at the School of Nursing at the University of KwaZulu-Natal, which will be kept for a period of 5 years before being shredded. Only the researcher and the research supervisor have access to the data collected and copies of relevant review documents.

#### Obtaining informed consent

The participants need to understand their contribution to the study and what the researcher is asking them to do. Thus, participation in this study was voluntary and details about the aims, research methods (what was done to collect data), objectives and the potential outcomes were explained. All participants completed a written informed consent form and each was given a signed copy for their personal records.

#### Providing the right to withdraw

During the research process, the participants were informed that participation was voluntary, they had the right to withdraw at any stage and not to answer any questions without this affecting their employment in any way [34].

## Results

The participants’ demographic details are followed by the results of the PIP questionnaire and the qualitative data from the semi-structured interviews of the five nurse managers**.**

### Demographic details

Of the 100 health workers who were invited to participate, 79 gave informed consent and completed the questionnaires, of whom 91.1% (*n* = 72) were women and 78.5% were from rural and semi-urban areas. Most (55.1%, *n* = 43) were aged 35–39 years, and 65.8% (*n* = 52) were MCHWs, while 7.7% (*n* = 6) were nurse managers, data managers and health facility MCHW supervisors (Table 1). Most (91.1%, *n* = 71) had worked in the maternal and newborn health care program for more than 4 years, over half (53.2%, *n* = 42) had completed their primary schooling and 22.8% (*n* = 18) had a university qualification (Table 1).

**Table 1:** Demographic characteristics (*N* = 79)

Characteristics	Variables	No.	%
Post (Area of work) (*n* = 79)	MCHW	52	65.8
	Health facility nurses/midwives in maternity ward	7	8.9
	Health facility Nurse manager	6	7.6
	Health facility Data manager	6	7.6
	Health facility MCHWs supervisor	6	7.6
	District hospital based MCHWs supervisor	2	2.5
Sex (*n =* 79)	Males	7	8.9
	Female	72	91.1
Settings (*n =* 79)	Rural areas	32	40.5
	Semi-urban	30	38.0
	Urban	17	21.5
Age group (*n =* 78)	25–29	11	14.1
	30–34	18	23.1
	35–39	43	55.1
	> = 40	6	7.7
Years of work experiences (*n =* 78)	< 1 year	4	5.1
	1–3 years	3	3.8
	> = 4 years	71	91.1
Education (*n =* 79)	No school	2	2.5
	Primary school	42	53.2
	Secondary School	17	21.5
	University	18	22.8

## Results of the quantitative PIP questionnaire

The results of the quantitative PIP questionnaire with 10 success areas were responded to by 77 participants (Appendix, Supplementary file 1, point 4). All the stakeholders (100%, *n* = 77/79) felt that the program mission (No. 1) was adequately addressed, while there was disagreement about the level of top management support (No. 2) (Table 2). Almost all the respondents agreed (98.6%, *n* = 74/79) that the program’s scheduled plans (No. 3) were in place and that client consultation (No. 4) had occurred (96.2%, *n* = 75/79). Human resources (No. 5) was regarded as an area of concern, with 18.7% (*n* = 14/79) indicating that they did not agree that this was adequately provided for. A few respondents (10.4%, *n* = 8/79) did not agree that the technical tasks (No. 6) were adequately available, while 98.6% (*n* = 75/79) agreed that client acceptance (No.7) was satisfactory, as indicated by their attending the program services.

**Table 2:** Stakeholder’s responses to the PIP questions

Key areas	Responses N. (%)					Mean	Std. Dev
	Strongly Disagree	Disagree	Neutral	Agree	Strongly Agree		
1. Program mission (*n =* 77)	0 (0)	0 (0)	0 (0)	47 (61)	30 (39)	4.39	.491
	0 (0)			77 (100)			
2. Top management support (*n =* 77)	5 (6.5)	9 (11.7)	3 (3.9)	50 (64.9)	10 (13)	**3.66**	1.059
	14 (18.4)			60 (77.9)			
3. Program scheduled plan (*n =* 75)	0 (0)	0 (0)	1 (1.3)	43 (57.3)	31 (41.3)	4.40	.52
	0 (0)			74 (98.6)			
4. Client consultation (*n =* 78)	0 (0)	0 (0)	3 (3.8)	36 (46.2)	39 (50)	4.46	.574
	0 (0)			75 (96.2)			
5. Human resources (*n =* 70)	5 (6.7)	9 (12)	5 (6.7)	47 (62.7)	9 (12)	**3.61**	1.064
	14 (18.7)			56 (74.7)			
6. Technical tasks (*n =* 77)	1 (1.3)	7 (9.1)	3 (3.9)	46 (59.7)	20 (26)	4	.889
	8 (10.4)			66 (85.7)			
7. Client acceptance (*n* = 76)	0 (0%)	0 (0%)	1 (1.3)	47 (61.8)	28 (36.8)	4.36	.509
	0 (0)			75 (98.6)			
8. Monitoring and feedback (*n* = 77)	0 (0)	6 (7.8)	1 (1.3)	43 (55.8)	27 (35.1)	4.18	.807
	6 (7.8)			70 (90.9)			
9. Communication (*n =* 77)	2 (2.6)	0 (0%)	0 (0)	48 (62.3)	27 (35.1)	4.47	.719
	2 (2.6)			75 (97.4)			
10. Urgent solutions for unexpected problems (*n =* 77)	27 (35.1)	9 (11.7)	1 (1.3)	30 (39)	10 (13)	**2.83**	1.559
	36 (46.8)			40 (52)			
Overall perception						40.21	4.797

The majority (90.9%, *n* = 70/79) agreed that monitoring and feedback (No.8) was done to monitor the progress of the program, while 97.4% (*n =* 75/79) agreed that adequate communication (No.9) was in place. Urgent solutions for unexpected problems (No. 10) was regarded as an areas of concern, with 46.8% (*n* = 36/79) indicating that they did not agree that there was a way to identify unexpected solution strategies. Three categories were below the mean value of 4 (agree, strongly agree), these being top management support (No. 2) (Mean = 3.66, SD = 1.059), human resources (No. 5) (Mean = 3.61, SD = 1.064) and urgent solutions for unexpected problems (No. 10) (Mean = 2.33, SD = 1.559) (Table 2), these being particular areas of concern (Table 2).

### Qualitative interviews

The same nurse managers who participated in the quantitative research were approached for interviews (Appendix, Supplementary file 2). Five of the six managers were available and participated in the interviews, of whom four were men and one was a women. All were between the ages of 30–40, had obtained a university education and had more than 4 year working experiences in the program. The nurse managers may be more knowledgeable about intervention care, as indicated by Munyiginya, Brysiewicz and Mill [35], as they had received extensive training on the initiative, and were responsible for training and advising other staff members under their supervision.

### Responses to the qualitative interviews

The content analysis entail categories being identified for each of the 10 PIP areas: the program mission, top management support, program schedule /plans, client consultation, human resource, technical task, client acceptance, monitoring and feedback, communication, urgent solution for unexpected problem that arise, as indicated in Table 3.

**Table 3:** 10 PIP question areas and resulting categories

Question	Category
1. How is the program mission communicated?	1: Provision of guidelines about maternal and newborn care program 2: Regular meetings for MCHWs and health providers 3: Health facility delivery 4: Continuing professional development 5 & 6: Community work (umuganda) and announcements in churches and markets.
2. What kind of top management support do you receive for the program?	1: Training, materials, money for home visits, supervision and leadership
3. What program schedule /plans are in place?	1: MCHWs visits to communities 2: Health facility staff visits to communities 3: Antenatal care service, 4: Immunization scheduled
4. How do you consult the clients of the program?	1: MCHWs home visits and a suggestion box
5. What human resources do you have in the program?	1: Shortage of health human resources and training needs
6. How is technology being used to implement the program?	1: RapidSMS an important communication tool
7. Have the clients accepted the services?	1: Communication between mother and MCHWs 2: Referrals between community, health facility and district hospitals
8. Is there monitoring and feedback to identify problems and address them on an ongoing basis?	1: MCHWs, health facilities staff and executive meetings at the health facility
9. How do you communicate with the health team and MCHWs, and the mothers and their family groups?	1: Refer and accompany mothers to health facilities, ambulance called and SMS used.
10. How do you manage unexpected problems that arise and how do you find urgent solutions?	1. Communication between mothers, MCHWs, MCHWs supervisors at health facility and District hospitals

### How is the program mission communicated?

Regarding communicating the program goal to the health providers of the program team, mothers and their family groups affected by the program work, the following categories emerged from the interviews (1) provision of guidelines about MNH care program, (2) regular meetings for MCHWs and health providers, (3) health facility delivery, (4) continuing professional development, (5) community work (umuganda) and (6) announcements in churches and markets.

### Category 1: provision of guidelines about maternal and newborn care program

At the central level, the Ministry of Health (Maternal and Child Health Unit, Community Health Desk) decentralized the MNH care guidelines to district hospitals, where relevant staff and community health supervisors implement programs that fall under their supervision. From the district hospitals, the supervisors have to distribute to the next level, the health facilities, and communicate with health facility management. The Health facility nurse managers communicate with the MCHWs by providing information and support. The health facility nurse managers described how the decentralized guidelines are disseminated to their staff and communities. The decentralization of maternal and newborn service guidelines and dissemination of information enables the health system to introduce material to all relevant staff down to the MCHWs, who are an important link for pregnant women, mothers and newborn community to access the health system at Health facility. The nurse managers explains:

The Ministry of Health provided guidelines at all levels on how to take care of mothers and newborns. The staff and the MCHWs communicate all the required information to the mother. **(Manager 2, rural)**

The country provides guidelines at all levels on how to take care of mothers and newborns. We hold regular meetings on a monthly basis … **(Manager 3, urban)**

### Category 2: regular meetings for MCHWs and health providers

Then nurse managers indicated that the MNHC program mission is communicated to the MCHWs and health providers through (a) regular meetings held at health facilities and in communities, (b) educative talks during ANC appointments and (c) MCHWs meeting at health facilities.

**a. Regular meetings**: at health facility, the nurse managers hold meetings once a month and communicates to the health providers any new programs or changes to existing ones. They may also hold short meetings in the morning before work starts, where they communicate instructions or information received from the upper health levels. The meetings are the channels of updating the health providers on maternal and the newborn health care program. The health facility nurse managers also update the community level service providers and recipients, including the MCHWs and families through the health education and campaign about new programs to reduce maternal and newborn deaths. The qualitative data supported these findings, with participants explaining:

We communicate the program goal to the health providers of the program team, MCHWs, mothers and their family group through regular meetings … **(Manager 4, semi-urban)**

**b. Educative talk or health education:** health facility staff provide information to pregnant women attending ANC services while they visit the health facilities. There are also peer group discussion facilitated by health providers who convey relevant information, including any service changes. The health facility community health supervisors have a scheduled monthly agenda to visit the MCHWs in their communities to update them on new programs or changes to the MNH care program. These activities contribute to updating the community and evaluating the pregnant women’s practices as part of the program.

We hold educative talks with the pregnant women during their peer group antenatal care services. We hold a meeting each month with MCHWs on how to improve the services for our clients. Each month, we go to the community to explain the program to the mothers**. (Manager 1, rural)**

**c. MCHWs meeting at Health facility:** the MCHWs attend monthly meetings at health facilities and submit monthly reports about what services they provided in their communities. The monthly meetings and MCHWs reports are a way of updating the health facility and district managers and supervisors about what services are being provided, given the resource limitations, and how problems that arise are addressed, thereby enabling the system to be monitored.

Regular meetings are held with MCHWs at the health facility and MCHWs provide information to the Health facility. MCHWs give us reports that contain necessary information; also, some explanations are provided to the staff regarding the importance of the program … We sensitize our clients every day before service provision. Health education talks are scheduled to explain the program to the mothers. Several times, we go in the community to explain the program to the mothers. **(Manager 3, urban)**

### Category 3: health facility delivery

The nurse managers explain to the MCHWs and health providers the importance of shifting delivery from home to health facilities to enable the women to access skilled birth attendants. The trained health providers on the MNHC program provide information to their colleagues and during community health education session. This is due to a concern about the high number of home delivery and the need to reduce the problems that could occur without the attendance of skilled health providers (nurse, midwife, medical doctor). One nurse manager said:

The goal is explained to the community by the MCHWs; they explain the importance of delivering at the Health facility. We explain to the staff and the people the importance of the program. **(Manager 2, rural)**

We explain to the MCHWs the importance of delivering at the health facility. Then the MCHWs mobilize the mothers to deliver at a health facility. **(Manager 5, semi-urban)**

### Category 4: continuing professional development

A nurse manager revealed that the national Ministry of Health offers, promotes and authorises ongoing training for health providers in their working place to improve their knowledge about maternal and newborn health care programs, and to ensure that they have the appropriate skills to provide the required services. This also takes the form of continuing professional development, with programmes being developed and provided by Ministry appointed staff. This requires the provision of training materials to enable instruction to be provided at the work place, including updating the health providers on MNHC guidelines, policies and program tools. A manager explains*:*

Top management offer continuing professional development (CPD) to health providers to allow them to improve their knowledge and explain the program to people, and we have enough and appropriate materials provided to perform our work correctly. **(Manager 5, semi-urban)**

### Category 5: community work (umuganda) and announcements in churches and markets

The nurse manager emphasized that the MNHC program mission is communicated to enable community sensitization (Umuganda), which is scheduled by the Rwanda Government once a month on the last Saturday at meetings, as well in churches and at market events. In Rwanda, umuganda is done once each month, where the local government administration ensure that work done by the community, such as hygiene around the households. After the activities, there is a talk about health or issues that the local administration wants to raise with the community. The church leaders also make announcements at the request of government, and allow a brief talk about the MNH program to be given during the service. In open markets, once agreed by the local government authority, public announcements are made using a microphone to communicate information about a relevant subject, such as the MNHC. The participant explains:

We communicate and explain the goal of the program in community administrative work (Umuganda). We use advertisements in churches and markets explaining the goals. **(Manager 1, rural)**

### What kind of top management support do you receive for the program?

The issues identified by the informants as part of top management support were training the MCHWs and health providers, and providing materials, money for the MCHWs to make home visits, supervision and leadership.

### Category 1: training, materials, money for home visits, supervision and leadership

The nurse managers revealed that the MCHWs and health providers received support from the Rwanda Ministry of Health in terms of training, materials, money for home visits and supervision. While these was support from top management, the nurse managers indicated that it was inadequate and inconsistent, making providing ongoing services difficult. The distribution of money depends on the Ministry of Health and its partners’ budgets, or what was made available for each program. They indicated the need to address issues related to training programs and materials, money for the MCHWs home visits and supervision in order to improve maternal and newborn health. The absence of this support made it difficult to provide the services, as indicated by a nurse manager:

Top management support us in the MNH care program by providing training for staff in order for them to improve their knowledge. Money for visits is provided, but not enough. Supervision and leadership on field are ensured. However, although everything is provided or supported, it seems not enough; we need more trainings, supervision, leadership, and money for home visits **(Manager 1, rural)*.***

We received training to improve the knowledge of the staff, and some materials are provided to perform the work, but still materials and training are not enough, especially training for newly recruited MCHWs **(Manager 2, rural)*.***

### What program schedule /plans are in place?

In the program, the following categories of activities were identified: (1) MCHWs visits to communities, (2) health facility staff visits to communities, (3) antenatal care service and (4) immunization scheduled.

### Category 1: MCHWs visits to communities

The nurse manager noted that the MCHWs are required to visit community members, with specific appointment schedules needing to be followed when a woman is pregnant and once she had delivered. This required them to have daily plans to follow-up and advise the women in their communities. The MCHWs also need to submit their monthly reports to the health facilities to enable them to be analysed and reported on. Communication between the MCHWs and health facility occurs via SMS using cell phones that are provided to enable them to have quick access to support. Health facility community staff in the MNCH program have a community visit once a month when information is shared and problems addressed. The villages consist of 50–100 households, with each MCHW providing services to approximately 30 households. While mother in their catchment areas should be seen once a month, it can be difficult for the MCHWs to keep to their schedules as they work voluntary and do not always received the financial support to visit the women and neonates who need their care. Nurse Manager explains:

We have a daily plan for MCHWs. For every activity done by MCHWs for mothers in the community, an SMS is sent to inform the health facility. **(Manager 4, semi-urban)**

We have a plan for community visits; it is once per months, and we give mothers advice about the Maternal and Newborn Health Care program. The MCHWs visit the mothers throughout their area to make sure the advice is followed. **(Manager 5, semi-urban)**

### Category 2: health facility staff visits to communities

The health facility staff visit communities in their catchment areas**,** with the MCHWs making up to 150 visits to the mothers per month in their catchment areas, with one pregnant women and mother being visited by a MCHWs at least once per month. They are required to provide a report once a month to the health facility, and are supervised by the health facility supervises once a semester. The schedule of looking after the mothers in their communities is known by the MCHWs, who inform their supervisors at the health facilities. The participant explained:

The maternal community health workers’ schedule include visiting every mother once a month in her catchment area. MCHWs make a report once a month. The schedule for MCHWs supervision is once per semester. Each month, we go into the field to explain the program to the mothers. (**Manager 1, rural)**

### Category 3: antenatal care service

The nurse manager noted that the MCHW are responsible for ANC maternal health within their communities to identify pregnant women, make regular follow-ups during the pregnancy and encourage delivery in health facilities where skilled health workers are available. Pregnant women are encouraged to attend the health facility for ANC services during the 12th weeks of gestation, with Rwandan culture often requiring that women hide their pregnancy until the 4th month, making it difficult to identify and address problems. The health facilities are open 24 h, where health providers work shifts to ensure continuous access to care. The health providers explain the ANC services they offers, and advise the mothers to access their services at any time to prevent complications and ensure a safe delivery. The nurse manager explains:

When the MCHWs know that a mother is pregnant, they do the follow-up and advise her to go for antenatal care service early at a health facility. The health facility is open 24 hours, ready for ANC services. We are available for pregnancy tests every day. Every month, we hold meetings to explain the maternal and newborn health care program to the mothers. The Community health workers visit mothers every day to make sure the advice is followed. **(Manager 2, rural)**

### Category 4: immunization scheduled

The participant revealed that the health facilities offer child immunization and ANC services as part of the MNH care program, these being scheduled at different time during the week. The staff at the different levels are able to communication with and remind the pregnant women and mothers with newborns to attend the relevant services on the appropriate dates. The participant explains:

We have a schedule of vaccination (immunization) and antenatal care services on different days throughout the week … (Manager 3, urban)

### How do you consult the clients of the program?

Regarding how the staff consulted the program clients as program users to find out what they thought was required, the nurse managers indicated that this was done during the MCHWs home visits and by providing a suggestion box at the health facility.

### Category 1: MCHWs home visits and a suggestion box

Home visit by MCHWs and a suggestion box were the channels of communication through which consultation occurred between the health facility, MCHWs and mothers. The participant revealed that the health facility staff communicate with clients (the users of health facilities services) by home visit undertaken by MCHWs, and at the health facilities, where suggestion boxes were provided for users to put their comments or recommendation about to the services received or required. This included issues related to the quality of services received by mother, pregnant women and their family as part of ongoing monitoring. The participant explains:

MCHWs visit the clients of our maternal and newborn health care program and send us a rapid SMS. **(Manager 4, semi-urban)**

We consult the clients of the program; we ask our stakeholders if they are satisfied with the services, we offer to them and ask them to tell us how to improve the services. In addition, they are free to use suggestions boxes to write to us telling us what they need for improvement. **(Manager 5, semi-urban)**

### What human resources do you have in the program?

The data obtained from the participants revealed the presence of MCHWs, A2 nurses, A1 and A0 midwives, MCHWs supervisors and data managers, but that there were shortages of some categories and additional training was needed.

### Category 1: shortage of health human resources and training needs

The participants revealed that the MNHC program had insufficient professionals and nonprofessional health workers, including A2 nurses, A1midwives, MCHWs and inadequate training*.*

### A2 nurses, A1 midwives, MCHWs and training needs

The participants revealed that there were insufficient midwives at each health facility, and that there was a need for more training about maternal and newborn health care program. The Ministry of Health introduced MCHWs into the program due to staff shortages at the health facilities, the intention being that they promote good health practices for ANC, delivery and PNC services. However, there were still insufficient health facility staff to provide the more specialized care that the community health workers could not provide, which the mother and newborn need around the time of birth. Quality care needs to be provided by the qualified health professionals and the MCHWs. The participants explained:

We have A2 nurses, and MCHWs who can read and write, and some of them hold at least 3 year secondary school certificate, and the MCHWs supervisor hold an A1 … In the MNH care program, we have maternal community health workers who have all the required materials, they are always provided with the materials that are needed. The maternal community health workers need to be trained … The people in this area move too much, change their dwelling too much; maternal community health workers cannot follow up on them. We don’t have any midwife; we are waiting for his or her appointment. **(Manager 4, semi-urban)**

We have one A1 midwife, two A2 nurses well trained and very competent … We do not have enough trained staff to take care of mothers and newborns; we have one midwife (A1) and nurses but their number is not enough … (**Manager 1, rural)**

### How is technology being used to implement the program?

The program has been greatly enhanced by implementing the RapidSMS system to exchange information between the staff at the different levels, with adequate staff being essential to ensure that the transfer of information works effectively.

### Category 1. RapidSMS an important communication tool

The nurse managers described how the use of the RapidSMS works, which is a platform that channels communication between community health workers, the ambulance system, health facilities staffs, and the central level (Ministry of health). Mobile phones, a charging device and airtime are provided for the MCHWs, the technology enabling message to be sent from them to the health facility staff, who send feedback with relevant information, with skilled staff being available to provide training on any changes to the RapidSMS system. However, there have been a number of challenges to its effective use, these being network problems and uncharged batteries, which can affected communication during emergencies, when information or ambulances need to be requested. The participants explains:

The Rapid SMS system uses a cell phone: the MCHWs send a message to the Ministry of Health, then feedback from Ministry of Health is provided through an SMS alerter, We use a telephone to send a rapid SMS to the Ministry of Health and the Ministry of Health sends feedback to acknowledge receipt. **(Manager 5, semi-urban)**

The MCHWs use a telephone to send a rapid SMS to the Ministry of Health providing information on pregnant women. When there is a danger, the MCHW sends a message to the Ministry of Health and the system informs the sector concerned. The system indicates to the MCHWs how to intervene in the case of difficulties. **(Manager 2, rural)**

### Have the clients accepted the services?

The nurse managers felt that the clients had accepted the program services, specifically the MCHWs in their communities, who were trusted and respected members and offered useful and relevant advice. Regarding the interaction between the mothers, MCHWs and health providers, the informants identified the following category as being important: (1) communication between mother and MCHWs and (2) referrals between community, health facility and district hospitals.

### Category 1: communication between mother and MCHWs

The managers revealed how the users of the maternal and newborn health care program, especially mothers, had accepted the services and found that communication with the MCHWs was an important link to their use at higher levels. The mothers communicate with MCHWs when they have a problem, which the MCHWs relay to higher level health providers for advice, which they feed back to the women. For those in rural areas who cannot easily access health services, the MCHWs provide an important intermediary step through which services and information can be accessed, as noted by a respondent:

When a problem arises in relation to the MNH care program, the MCHWs send us information by phone. Then, the staff works with the MCHWs who has sent the information and share the information with us (HF manager). The health facility sends back the information required to the maternal community health workers advising them on how to solve the problems**. (Manager 4, semi-urban)**

When mothers experience problems in the community, they inform the MCHW on duty who has been selected in the village; then MCHW intervenes, solves the problem and communicates with the health facility. However, when the problem is hard for them to solve the MCHW sends the mother to the health facility. **(Manager 5, semi-urban)**

### Category 2: referrals between community, health facility and district hospitals

The nurse managers described the acceptance of health facility services through the use of referral forms as a means of communicating information, problems and solution between the different levels of care. The referral form support the transfer of patients to higher levels of care, from the MCHWs to the health facility and district hospital when more specialized input is needed. The results of the larger study found that 82.3% (*n* = 65/79) of pregnant women were given referral notes from the MCHWs to present at health facilities, while 53.2% (*n* = 42/79) are given referral notes to give back to the MCHWs when they returned home. There is a need to improve the number of referral notes provided by the health facilities to the MCHWs for continuous of care to be ensured.

The staff work closely with MCHWs who deal with the problem; if they cannot solve the problem, a document is written and sent to the health care center. The health center sends required information to the MCHWs. **(Manager 2, rural**)

The mothers inform MCHWs who deal with the problem. Sometimes the MCHWs send the mother to a health facility with a document explaining the case. The health facility provides care to the mother and sends required information to the MCHW. This means that the mother may come to the center so that we deal with the problem. In case the problem cannot be resolved at health facility level, the health facility sends the mother to the hospital. **(Manager 1, rural)**

### Is there monitoring and feedback to identify problems and address them on an ongoing basis?

From the responses related to monitoring the progress of the program implementation and providing feedback to the program team, the following meetings were held monthly: (1) MCHWs, staff and executive managers.

### Category 1: monthly MCHWs, staff and executive meetings at health facility

The participants commented on the regular meetings conducted to monitor program progress and improve feedback to the program team to enhance the MNH care services at all levels. The MCHWs were required to attend monthly meetings to raise problems, address areas of concern, and obtain feedback from previous discussions. Meetings were held monthly with all the health facility-based staff involved in the program to address local issues and those related to referrals between the MCHWs and district hospitals. The managers from the district hospitals and health facilities held executive meetings to address system and process issues, as well as those raised during the MCHW and staff meetings. All the meetings were focused on monitoring the implementation of the program, and providing feedback to all health providers in the team to ensure overall coordination of activities and information, and to address problems as they arose. The participant explains:

We conduct regular meeting to monitor the progress and improve the feedback to the program team at the health facility. The monthly meeting with all MCHWs is chaired by the HF manager. **(Manager 4, semi-urban)**

The health facility helps the maternal community health workers to monitor the progress. We hold monthly meetings with the staff and monthly meetings with executive community general assembly. **(Manager 5, semi-urban)**

We hold a meeting every month with maternal community health workers to discuss the progress of the program, and a monthly meeting with the staff to talk about the activities running in health facility and find a solution to any problem raised. **(Manager 1, rural)**

We hold regular meetings with health providers; we can update them on the program as well as the importance of the program … **(Manager 5, semi-urban)**

### How do you communicate with the health team and MCHWs, the mothers and their family groups?

Regarding communication between the program teams and their clients, the following issues were identified by the informants: (1) refer and accompany mothers to health facilities, in emergency cases, the ambulance is called, and the Rapid SMS system is a rapid and effective method.

### Category 1: refer and accompany mothers to health facilities, ambulance called and RapidSMS used

When pregnant women, mothers and newborn have issues that cannot be addressed by the MCHWs, they inform the health facility by sending an SMS or calling the nurse manager, who assists with advice or information. The health facility may sends an ambulance or the MCHWs refer and accompany mothers to health facilities. This often occurs when emergencies arise in communities that the MCHW cannot solve on their own, or do not have the authority to make decisions about. Most health facilities in Rwanda do not have ambulances, these being located at the district hospitals, with the former having to call the latter to access this service, which may be in use in anther district and results in long delays and people having to find other means of transport.

MCHWs refer pregnant women for ANC, accompany women to health facilities for delivery, and refer mothers and newborns for PNC … **(Manager 3, urban)**

Another participant said

… the MCHWs do the follow-up of mothers and babies at community level, with the health facilities, and in case of emergency, the ambulance is called … **(Manager 2, rural)**

MCHWs were given mobile phones that enabled them to collect and receive real-time data on maternal, neonatal … in their catchment areas by SMS **(Manager 2, rural)**

### How do you manage unexpected problems that arise in the program and how do you find urgent solutions?

Regarding managing unexpected problems that arise in the program, the nurse managers indicated the importance of communication between the MCHWs, mothers and managers at health facility and district levels, and where possible, to collaborate with community leaders.

### Category 1. Communication between mothers, MCHWs, MCHWs supervisors at health facility level and district hospital

The nurse managers indicated the need to have effective and reliable means of communication to address unexpected problems, and to consult with staff and community members to provide appropriate solutions. Where communication is difficult via cell phones to health facility staff, it may be necessary to consult locally, for example, to secure transport to a health facility during an emergency. They indicated the need to have good communication before such situations occur, and to include community leaders and family members in maternal and neonate health issues as part of community education to ensure their support and participation when unexpected situations arose. While it is not possible to anticipate all the problems that might arise, it was possible to develop strategies to address the common one that arose locally and in other areas as a way of being prepared for unexpected situation. When cell phone communication was possible, the MCHWs contacted the health facility managers or district supervisors for assistance with urgent situations, as they had access to more specialized staff and services, such as ambulances, and could authorize actions that the MCHWs could not. The nurse manager explains:

When an unexpected problem arises in the program, the MCHW supervisor at health facility level follows it up and inform us (nurse managers). Also, the District hospital MCHWs supervisor can help us when we have a problem. **(Manager 4, semi-urban)**

Emergency problems are dealt with at District hospital level. People may bring the mother at Health facility using the traditional transportation mode. If possible, and in case of emergency, we call for an ambulance. **(Manager 1, rural)**

The health facility resolves the problem, but when it is difficult to resolve the problem, we refer the mother to a higher level, the district hospital. (**Manager 2, rural)**

## Discussion

This study explored the stakeholder’s perceptions of the implementation of the Rwandan maternal and newborn health care program to identify areas for improvement. The adapted 10 critical success areas for Program Implementation Profile (PIP) among existing maternal and newborn health care program in Rwanda were applied: (1) project mission, (2) top management support, (3) program schedule/plan, (4) client consultation, (5) human resource, (6) technical tasks, (7) client acceptance, (8) monitoring and feedback, (9) communication and (10) urgent solutions for unexpected problem.

The convergent parallel mixed methods entailed the use of both quantitative and qualitative data, with the two data sets being analysed separately but combined for the interpretation and discussions of the results. The findings of the quantitative results from the PIP questionnaire showed that the three of the 10 areas need attention, these being top management support, human resource and urgent solutions for unexpected problem to improve the programs’ implementation and reduce maternal and newborn deaths, these being discussed further.

While the quantitative data indicated the number of participants and the extent of their dissatisfaction with top management, the qualitative data provided the details of the components that they were dissatisfied with. Further research is required to establish if there are differences in the issues that need to be address between rural, semi-urban and urban areas. As most district hospitals are located in urban areas, the urban health facilities relying on them for support may have better access to services than rural ones.

### Top management support

In this study, top management support relates to the willingness of senior health services management to provide the necessary resources and exercise their authority/power to ensure that the maternal and newborn health care program is implemented effectively. The findings showed diverse opinions about the level of top management support (**No. 2**), with almost one fifth (*n* = 14/77, 18.4%) indicating their dissatisfaction. In this study, the satisfaction was based on the key performance requirements for top management support, which were allocating adequate money; quantifying total resources required for implementation, especially for the program activities, and providing training, MCHWs equipment and supplies.

The interviews showed that the managers understood the resources required (time, manpower, equipment and training) to implement this program. They were aware of the need to provide adequate and appropriate training for staffs, health providers and MCHWs; distribute money for home visits and materials, and ensure supervision and leadership in order to reduce maternal and newborn mortality. While such support was offered, the questionnaire indicated that the participants felt that it was insufficient. The interviews indicated that having only one ambulance for emergency transfers per district hospital, which included several health facilities, and only one delivery room in each health facility, was inadequate to meet the needs of those requiring such services.

The Rwandan maternal and newborn health care program was initiated with the costs being shared between the MOH and development partners for various aspects, including training. Likewise, in many low- and middle-income countries, non-government organizations (NGOs) supplement the formal health system. These agencies are often relatively new, have unstable funding streams, inadequate ratios of staff members to the number of family served, and the donor /funder priorities guide the local programming, which results in many programs not being effective [36]. This finding was supported by studies in Asia and African LMIC, where NGOs initially provided services, but then struggled to feasible implement competent programs [36].

A theme identified in this study, the need for more training, materials, money for home visits, supervision and leadership, has been identified elsewhere in Rwanda [37]. In addition, the lack of refresher trainings/mentorship has also being reported as a barrier to applying new knowledge and skills among health providers [38]. This has been identified as an issue in a study in Uganda, which indicated that supervision helped district health managers to identify and address maternal and newborn services-delivery gaps [39]. In South Africa, an evaluation of the CHWs training revealed that most demonstrated the necessary skills for referrals to prevent complications, and to provide care for newborns and their mothers at home immediately after discharge from health care centres [40].

### Human resources

In this study, insufficient human resources was identified as an area needing improvement by the participants, their responsibilities being recruitment, selection and training of the necessary personnel for the maternal and newborn health care program. Human resources **(No. 5)** was regarded as an area of concern, with 18.7% (*n* = 14) indicating that they did not agree that this was adequately provided for. A study conducted in Rwanda showed that health facility and community staff had insufficient training [15], which challenged the effectiveness of the MNH program, as many staff members are non-healthcare professionals who lack the basics healthcare education background, such as MCHWs [41]. This was similar to a study in South Africa, where the MCHW had a low levels of education, many not having completed their school education [42]. Aside from the issue of training, the findings for this study also reiterate those elsewhere on the issue of shortage of qualified health staff [43].

The Rwanda Maternal Newborn and Child Strategic Plan 2018–2024 noted the human resources challenges relating to enabling women to giving birth in health facility and being assisted by skilled providers, with quality of care being an ongoing problem. A focus on training additional skilled, competent providers, especially midwives, is an issue that needs to be addressed [15]. The findings of this study showed that the MNHC program needs to increase the number of skilled health providers and provide ongoing training. To improve access to health services by bringing services closer to the communities and addressing the shortage of the health care work force, the MOH endorsed the program of nominating MCHWs from their communities. However, the quality of the comprehensive package of services remains a challenge, including the limited capacity of the MCHWs; insufficient resources to sustain routine community health activities, such as refresher training; and the need to reinforce supply systems, purchase equipment, upgrade infrastructures in order to deliver more health services to communities [17].

Due to country–specific issues related to MCHWs, the selection of community health workers is often based on nomination rather than professional competence or capacity, and therefore requires extensive training. Organizations tend to identify socially competent and committed individuals who are good problem solvers and train them in the specific skills needed. Having likable, committed, smart staff who are invested in the MCH’s mission makes daily operations easier and offers the opportunity to deliver a high–quality program [36]. High quality maternal and child health programs are needed to meet the global Sustainable Development Goals of ending poverty (Goal 1) and hunger (Goal 2), improving health and well-being (Goal 3) and reducing inequity Goal 10) by the year 2030 [36].

The nurse managers raised the issues of a shortage of suitably qualified health care providers in the program, with each health facility having only one midwife with an A1 qualification, while some nurses had A2 certificates, which is regarded as an inadequate qualification to provide quality maternity services in the Rwandan health system, these nurses being advised to upgrade their certification. Furthermore, they indicated the need to increase the number of midwives at the health facilities, as one was not enough for both the day and night shifts. Another study conducted in Rwanda also recommended an equitable health workforce, with training being essential to address the determinants of poor health and reduce health inequalities [44]. A review from Africa and Asia identified CHWs as providing MNH services in Bangladesh, Indian, Kenya, Malawi and Nigeria, and showed that they face pressure to provide services beyond their scope of practice, specifically during emergencies. There was also a tendency in some settings to focus CHWs on facility–based roles at the expense of their traditional community–based responsibilities [45]. Thus, there is a need to review and revise their scope of practice to reflect the varied duration of training and in-country legislation [45].

The quantitative data from the PIP questionnaire indicated that human resources were a problem, with the nurse manager interviews providing details about the areas of greatest concern, indicating what needs to be done to improve MNH services in Rwanda. The human resources issues were insufficient midwives and training, with a call for advocacy for maternal and newborn health of sufficient quality to meet the SDGs.

### Urgent solutions for unexpected problem

In the current study, urgent solutions for unexpected problem was highlighted (**No. 10**) as an area of concern, with 46.8% (*n* = 36) indicating that they did not feel that the staff were equipped to address this adequately. The findings are similar to that of Rwanda’s Ministry of Health Strategic Plan 2018–2024, which showed that this may be due to the inadequate healthcare workforce, this being one midwife per 4064 women aged 5–49, one nurse per 1094 people, one doctor (general practitioner and specialists) per 10.055 people, while the SDGs recommended 4.45 nurses, midwives and doctor per 1000 population [15, 46]. Similarly, Ghana’s health system experienced constraints, such as the availability of staff, essential medicines, supplies and equipment, and management issues (including leadership and interpersonal relations among staff). Issues related to poor cellphone network coverage, lack of sufficient ambulances, inexperienced MCHWs, lack of midwives at health facilities, poor road infrastructure, and cultural preferences could result in problems arising over which the health staff have not control, but which affect access to and the quality of services. Problem solving in resource constrained settings needs to be included as part of the training, and alterative systems put in place to take into consideration the common constrain that the staff may face. There needs to be broader support for the MNH by developing effective interventions to address the immediate as well as more long-term challenges that influence decision–making [47].

In Ethiopian, low maternal health services utilization was related to a range of issues, such as women’s socio-demographics, cultural and communal factors, limited access to health facilities, and poor quality of care in health facilities. These complex and interlinked factors can be characterised by three delays model that affect emergencies [7]: 1) delay in deciding to seek care, 2) in reaching the health facility, and 3) in receiving quality care once at the health facility. The Ethiopian Government expanded their health extension program into communities, improved community ownership of health services and scaled-up best practices to overcome delay 1 [7]. In responding to delay 2, they introduced an innovative free ambulance services in every rural districts that are available on a community basis to transfer any woman in labour or experiencing other obstetric difficulties to the appropriate health facility [7]. These measures helped to address unexpected problems that can arise at any time during a pregnancy, or once a child has been born and needs emergency care. Inadequate care at a health facility can result in a case needing to be transferred upward to a district facility, which takes time and resources, such as ambulances, and can compromise the health outcome of the patients.

Finding appropriate urgent solutions entailed collaborating with community leaders, using traditional transport and communicating with the mothers and their families to alert them to possible issues to find locally relevant ways to address them. Some solutions could be time-consuming and result in delays in getting to health facilities to address the emergencies. This was reported in a study in Rwanda, which noted an inadequate workforce at health facilities, which prevents the more experienced staff from being able to undertake home visits, and that when problems arose among the maternal and newborn staff at health facilities, the possible solutions were not discussed among the staff due to these shortages [48]. Many maternal deaths (33 to 50%) can be attributed to insufficient staff, poor ANC and the inadequate recognition of high-risk pregnancies, as well as the lack of early detection of risk factors that indicate the need to refer to higher levels of care [48]. The same authors reported that in Rwanda, 69% of deliveries were assisted in health facilities, with only 18% receiving PNC by skilled health workers due to the high workload of the limited number of staff [48]. This study indicates the need to explore the complexity of issues faced by the MNC program to be able to address the unexpected issues, such as improving top management’s ability to provide qualified human resources, who will be able to detect and handle the unexpected problems.

This article has potential implications for the body of knowledge that shapes policies that direct evidence-based maternal and neonatal health care practice in Rwanda. The study had highlighted the need for ongoing monitoring and evaluation using a comprehensive and standardized tool, such as the PIP, to enable managers to effectively implement the maternal and newborn health care program across the country. Differences between rural and urban areas need to be identified and addressed, as well as the effect of limited resources and constraints that are beyond the control of the Ministry of Health. Once it has been implemented, ongoing monitoring is essential to ensure that the standards and systems are maintained, and against which any change in service use and maternal and neonate morbidity and mortality can be evaluated.

### Limitations

Regarding its limitations, the sample size was small and the study was conducted in only three district hospitals, which means that the findings cannot be generalized to all settings. While many MCHWs participated, very few staff at the health facilities were included, which may have affected the findings. In addition, this is the first time that the PIP instrument was used in Rwanda, and it may need modifications to ensure that it is appropriate for the county’s health care context.

## Conclusions

Implementing an effective maternal and newborn health program in Rwanda is an essential part of reducing morbidity and mortality in the resource-constrained country. Since its roll-out in 2008, the MNH has been implemented with varying levels of success around the country, indicating the need for ongoing monitoring to ensure that its weaknesses are understood and addressed, and its strengths are acknowledged and replicated. While most of the participants generally agreed that the key areas were being adequately dealt with, the three areas of concern of top management support, human resources and urgent solutions for unexpected problems needs further investigation to establish the reasons for these responses, and to find ways to improve them, as they may well be affecting the provision and uptake of maternal and newborn health care program services.

## Supplementary Information

**Additional file 1.**

**Additional file 2.**

## Data Availability

The datasets analysed during the current study are available from the corresponding author on reasonable request.

## Abbreviations

ANC: Antenatal Care
Au: African Union
BREC: Biomedical Research Ethics Committee
CARMMA: Campaign for Accelerated the Reduction of Maternal, Newborn and Child Mortality in Africa
CB- MNH: Community Based -Maternal and Newborn Health
CHWs: Community Health Workers
CPD: Continuing Professional Development
EMoC: Emergency obstetric Care
HB- MNCP: Home-Based Maternal and Neonatal Health Care Package
HEARD: Health Economics and HIV/ AIDS Research Division
LB: Live Births
LMIC: Low Middle Income Country
MCHIP: Maternal and Child Health Integrated Program
MCHW: Maternal Community Health Workers
MDGs: Millennium Development Goals
MMR: Maternal Mortality Ratio
MNCH: Maternal, Neonatal and Child Health
MoH: Ministry of Health
NGOs: Non-Government Organizations
PHC: Primary Health Care
PIP: Project Implementation Profile
PMI: Project Management Institute
RNEC: Rwanda National Ethics committee
SDGs: Sustainable Development Goals
SMS: Short Messages Service
SSA: Sub- Saharan Africa
USAID: United States Agency for International Development
