# Supplementary material for: Stakeholders perceptions regarding implementing maternal and newborn health care programs in Rwanda
Source: BMC Health Serv Res. 2021 Aug 11;21:796. doi: 10.1186/s12913-021-06824-3 (PMC8359551; doi:10.1186/s12913-021-06824-3)
Supplement: Supplementary file 2 — Additional file 2. [file 12913_2021_6824_MOESM2_ESM.docx]

**Identification:**

**Date: Time: Settings:**

**Designation: Nurse Manager**

**Sex: ………..**

**Age group: 1.** 25-29**…..; 2.** 30-34**…….; 3.**35-39**….; 4.** 40

**Year of experiences:…………**

**Education:……………….**

**Semi –structured interview guide**

**For managers : Critical success factors of program implementation extent. (**Slevin,D.P. & Pinto,J.K. 1986)

1. What problems do you face with regard to the maternal and newborn health care program mission?

2. What kind of top management support do you receive for the program?

3. What program schedule /plan do you have?

4. How do you consult the clients of the program?

5. What kind of Human resources do you have in the program especially regarding their qualification?

6. How is the RapidSMS-MCH system being implemented?

7. How do Mother, MCHWs, health provider interact when problems or questions arise relating to the program?

8. How do you conduct regular meetings to monitor program progress and improve the feedback to the program team?

9. How do you communicate the program goal to the health providers of the program team and MCHWs, mothers and their family groups affected by program work?

10. How do you manage unexpected problems that arise in program and how do you find urgent solutions.
